# Synthesizing Polyaniline With Laccase/O_2_ as Catalyst

**DOI:** 10.3389/fbioe.2019.00165

**Published:** 2019-07-10

**Authors:** Peter Walde, Keita Kashima, Gordana Ćirić-Marjanović

**Affiliations:** ^1^Laboratory for Multifunctional Materials, Department of Materials, ETH, Zurich, Switzerland; ^2^Department of Chemistry and Bioengineering, National Institute of Technology, Oyama College, Oyama, Japan; ^3^Faculty of Physical Chemistry, University of Belgrade, Belgrade, Serbia

**Keywords:** aniline, enzyme, lignin, laccase, peroxidase, polymerization, polyaniline, template

## Abstract

The polymerization of aniline to polyaniline (PANI) can be achieved chemically, electrochemically or enzymatically. In all cases, the products obtained are mixtures of molecules which are constituted by aniline units. Depending on the synthesis conditions there are variations (i) in the way the aniline molecules are connected, (ii) in the average number of aniline units per molecule, (iii) in the oxidation state, and (iv) in the degree of protonation. For many possible applications, the synthesis of electroconductive PANI with *para*-N-C-coupled aniline units in their half-oxidized and protonated state is of interest. This is the emeraldine salt form of PANI, abbreviated as PANI-ES. The enzymatic synthesis of PANI-ES is an environmentally friendly alternative to conventional chemical or electrochemical methods. Although many studies have been devoted to the *in vitro* synthesis of PANI-ES by using heme peroxidases with added hydrogen peroxide (H_2_O_2_) as the oxidant, the application of laccases is of particular interest since the oxidant for these multicopper enzymes is molecular oxygen (O_2_) from air, which is beneficial from environmental and economic points of view. *In vivo*, laccases participate in the synthesis and degradation of lignin. Various attempts of synthesizing PANI-ES with laccase/O_2_ in slightly acidic aqueous media from aniline or the linear aniline dimer PADPA (*p*-aminodiphenylamine) are summarized. Advances in the understanding of the positive effects of soft dynamic templates, as chemical structure guiding additives (anionic polyelectrolytes, micelles, or vesicles), for obtaining PANI-ES-rich products are highlighted. Conceptually, some of these template effects appear to be related to the effect “dirigent proteins” exert in the biosynthesis of lignin. In both cases intermediate radicals are formed enzymatically which then must react in a controlled way in follow-up reactions for obtaining the desired products. These follow-up reactions are controlled to some extent by the templates or specific proteins.

## The Participation of Laccases in the *In Vivo* Synthesis and Degradation of Lignin

**Figure 1:**
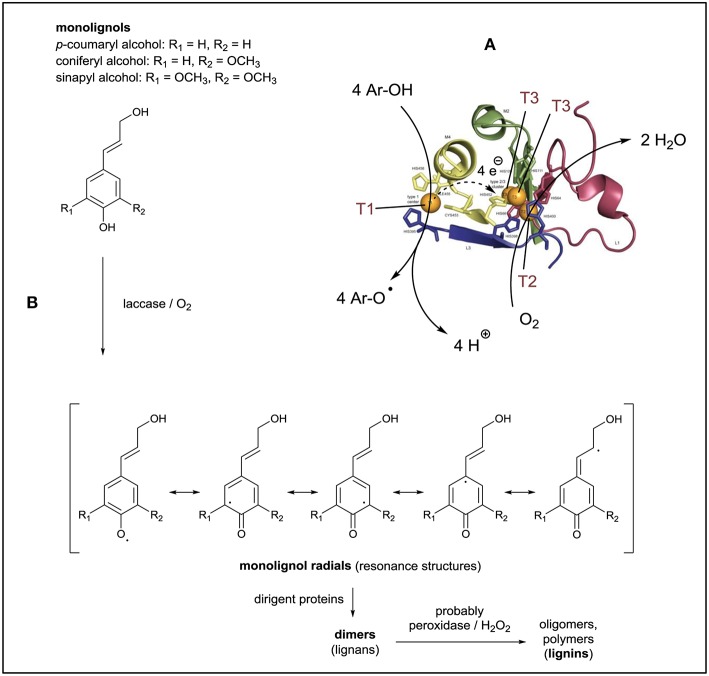
Laccase/O_2_-catalyzed reactions in connection to the synthesis of lignin. **(A)** The crystal structure of the two active sites of *Trametes versicolor* laccase illustrates the four copper ions which participate in the reaction. At the T1 site, the substrate molecules are oxidized, followed by electron transfer from T1 to the trinuclear copper cluster (TNC) where reoxidation takes place with dissolved O_2_. Reproduced and modified from Sirim et al. (2011). **(B)** Monolignols as natural substrates of plant laccases, showing the resonance structures of the intermediates obtained from the one-electron oxidation of the monolignols. The initial follow-up reactions are assisted by dirigent proteins and lead to the formation of lignans, which upon further oxidative oligomerization yield lignin.

Laccases (EC 1.10.3.2) are metalloenzymes that form a subfamily within the superfamily of multicopper oxidases found in fungi, higher plants, bacteria, and insects (Solomon et al., 1996, 2014; Sirim et al., 2011). Laccases have four copper ions that constitute two spatially separated active sites. One active site is formed by one copper ion (type 1, abbreviated as T1), the other by a trinuclear copper cluster (TNC) consisting of T2 (one copper ion) and T3 (two copper ions). Laccases oxidize a broad range of substrates at T1 in a one-electron oxidation reaction, for example the oxidation of phenol derivatives (Ar-OH) to the corresponding phenoxy radicals (Ar-O^•^), whereby the electron which is released from the substrate is transferred *via* a His-Cys-His tripeptide from T1 to the TNC where dissolved molecular oxygen (O_2_) is bound, activated and reduced in a four-electron reduction (Bertrand et al., 2002; Morozova et al., 2007a; Solomon et al., 2014; Jones and Solomon, 2015). In one catalytic laccase cycle, four substrate molecules (e.g., Ar-OH) are oxidized at the expenses of one molecule O_2_, yielding in the case of Ar-OH four phenoxy radicals (Ar-O^•^) and two water molecules as side products: 4 Ar-OH + O_2_ → 4 Ar-O^•^ + 2 H_2_O (Figure 1A). Ar-O^•^ then undergoes follow-up reactions. The copper ion at T1 is responsible for the blue color of laccases, with λ_max_ ≈ 600 nm and a molar absorption ε_≈600_ ≈ 5,000 M^−1^ cm^−1^ (Solomon et al., 1996). Although these “blue laccases” (Morozova et al., 2007a) are the ones we think of if laccases are mentioned, white and yellow laccases also exist as modifications of blue laccases, lacking the absorption band at λ ≈ 600 nm (Leontievsky et al., 1997; Agrawal et al., 2018).

In plants, laccases participate in the *biosynthesis of lignin*, which is a complex, branched, heterogeneous, and water-insoluble, amorphous polymer (Roth and Spiess, 2015). Due to the insolubility of lignin it is difficult to determine its molar mass. Reported values for number or weight averaged molar masses are in the range of a few thousands to tens of thousands grams per mol (with a rather high polydispersity), depending on the lignin source, the pre-treatment conditions, and the isolation method (Tolbert et al., 2014). Lignin is formed from basically three hydroxycinnamyl alcohols (so-called monolignols): non-methoxylated *p*-cumaryl alcohol, mono-methoxylated coniferyl alcohol, and di-methoxylated sinapyl alcohol (Figure 1B). These monolignols are polymerized through radical couplings of their oxidized forms, resulting in an entangled polymeric network of phenolic and non-phenolic linking units (Barros et al., 2015; Roth and Spiess, 2015; Munk et al., 2018). Plant laccases participate at least in the very first step of lignin formation (Sterjiades et al., 1992; Solomon et al., 2014), which is the laccase/O_2_-catalyzed oxidation of monilignols to the corresponding monolignol phenoxy radicals. Since the unpaired electron can be located on different atoms of the radicals (see the different resonance structures in Figure 1B), the next step of the reaction, the coupling of two radicals to form a dimer (called lignan), leads to products which can have very diverse constitutions and different configurations. This is due to the various radical-radical coupling possibilities. Follow-up reactions, in which peroxidases and hydrogen peroxide (H_2_O_2_) are involved as well (Solomon et al., 2014; Barros et al., 2015), lead to the formation of oligomers and the final polymeric product (lignin). There is increasing evidence that the *in vivo* coupling of the phenoxy radicals which are produced from monolignols by oxidative enzymes like laccases (Figure 1B) is controlled, at least to some extent, through interactions with so-called “dirigent proteins” (Davin and Lewis, 2005). Whether these directing proteins are true enzymes with catalytic activity or not, needs to be clarified (Gasper et al., 2016). In any case it is evident that “dirigent proteins” direct regio- and stereoselectivity in bimolecular phenoxy radical coupling during lignan biosynthesis, and they may play the same role during the follow-up steps leading to the formation of lignin (Pickel and Schaller, 2013; Kim et al., 2015; Guerriero et al., 2016; Paniagua et al., 2017).

Apart from the involvement of plant laccases in the complex biosynthesis of lignan and lignin, laccases which are released from wood-rotting fungi together with other ligninolytic enzymes (including lignin peroxidase and Manganese-dependent peroxidase) (Wong, 2009; Janusz et al., 2017; Martínez et al., 2018) also participate in the *degradation of lignin* present in the wood on which the fungi live (e.g., Baldrian, 2006; Giardina et al., 2010; Roth and Spiess, 2015). For an efficient *in vitro* degradation of lignin the removal of degradation products seems to be important for preventing a re-synthesis (Roth and Spiess, 2015; Munk et al., 2018). Furthermore, the efficiency of the laccase for degrading lignin can be improved by using so-called mediators (organic or inorganic compounds or metal ions), which can reach the T1 site of the laccase and which have redox potentials that are lower or comparable to the redox potential of the laccase involved, such that these mediator molecules are oxidized by the laccase (Bourbonnais and Paice, 1990; Morozova et al., 2007b; Roth and Spiess, 2015; Longe et al., 2018). In this case, fungal laccases oxidize the mediator molecules, and the oxidized mediator molecules oxidize lignin, which can occur *via* different mechanisms, finally resulting in lignin degradation. The presence of mediator molecules seems to be essential for the complete degradation of lignin by laccases; without mediators, only the breaking of bonds in phenolic model compounds of lignin is catalyzed, for non-phenolic subunits, the use of mediators appears a must (Munk et al., 2015).

## The Use of Laccases for *In Vitro* Oligo-and Polymerization Reactions

Laccases, in particular the ones from *Trametes* fungi with their high oxidation potentials of ~0.78 V vs. NHE at the T1 site (Morozova et al., 2007a) and solvent exposed, about 5–8 Å deep, hydrophobic binding site near T1 (Solomon et al., 2014), have a very broad range of accessible substrates (Xu, 1996; Baldrian, 2006; Tadesse et al., 2008; Strong and Claus, 2011; Reiss et al., 2013). This allows for mediator-free *in vitro* applications which go beyond laccase-catalyzed oxidative transformations of physiological substrates. One example is the oxidation of aniline (Ph-NH_2_) (Reiss et al., 2013) despite its relatively low standard oxidation potential of −1.0 V (${\text{E}}_{\text{ox}}^{\circ }$ (Ph-NH_2_/Ph-N${\text{H}}_{2}^{•+}$) = −1.0 V vs. NHE) (Jonsson et al., 1994). If laccase-catalyzed transformations of non-physiological substrates in the presence of mediators are considered as well, it is not surprising that laccases are recognized as very valuable biocatalysts for many commercial and research applications in various areas (e.g., Riva, 2006; Rodríguez Couto and Toca Herrera, 2006; Kunamneni et al., 2008; Mikolasch and Schauer, 2009; Kudanga et al., 2011; Hollmann and Arends, 2012; Polak and Jarosz-Wilkolazka, 2012; Sousa et al., 2013; Pezzella et al., 2015; Mate and Alcalde, 2016; Upadhyay et al., 2016; Cannatelli and Ragauskas, 2017; Yang et al., 2017;Slagman et al., 2018).

Applications of laccase-catalyzed *in vitro* reactions include the laccase/O_2_-mediated syntheses of polymeric (or oligomeric) products from methyl methacrylate or styrene (with acetylacetone as mediator, see Tsujimoto et al., 2001), acrylamide (Ikeda et al., 1998), phenols (Mita et al., 2003; Marjasvaara et al., 2006; Sun et al., 2013; Su et al., 2018a, 2019a), pyrrole (Song and Palmore, 2005; Junker et al., 2015), dopamine (Tan et al., 2010; Li et al., 2018), 3,4-ethylenedioxythiophene (Shumakovich et al., 2012b; Vasil'eva et al., 2018), or various arylamines (Ćirić-Marjanović et al., 2017; Zhang T. et al., 2018; Su et al., 2019a,b). With respect to the latter type of monomers, the focus often was—and still is—on the synthesis of oligo- or polyaniline (PANI) from aniline (Karamyshev et al., 2003; Vasil'eva et al., 2007, 2009; Streltsov et al., 2008, 2009; Shumakovich et al., 2010, 2012a, 2014; Leppänen et al., 2013; Zhang et al., 2014, 2016; Zhang Y. et al., 2018; Junker et al., 2014a; de Salas et al., 2016; Su et al., 2018b) or from *p*-aminodiphenylamine (PADPA), the linear *para* N-C-coupled aniline dimer Shumakovich et al., 2011; Junker et al., 2014b; Janoševic Ležaić et al., 2016; Luginbühl et al., 2016; Kashima et al., 2018;Kashima et al., 2019).

## Laccase/O_2_-catalyzed Synthesis of Polyaniline (PANI) and the Role of “Templates”

**Figure 2:**
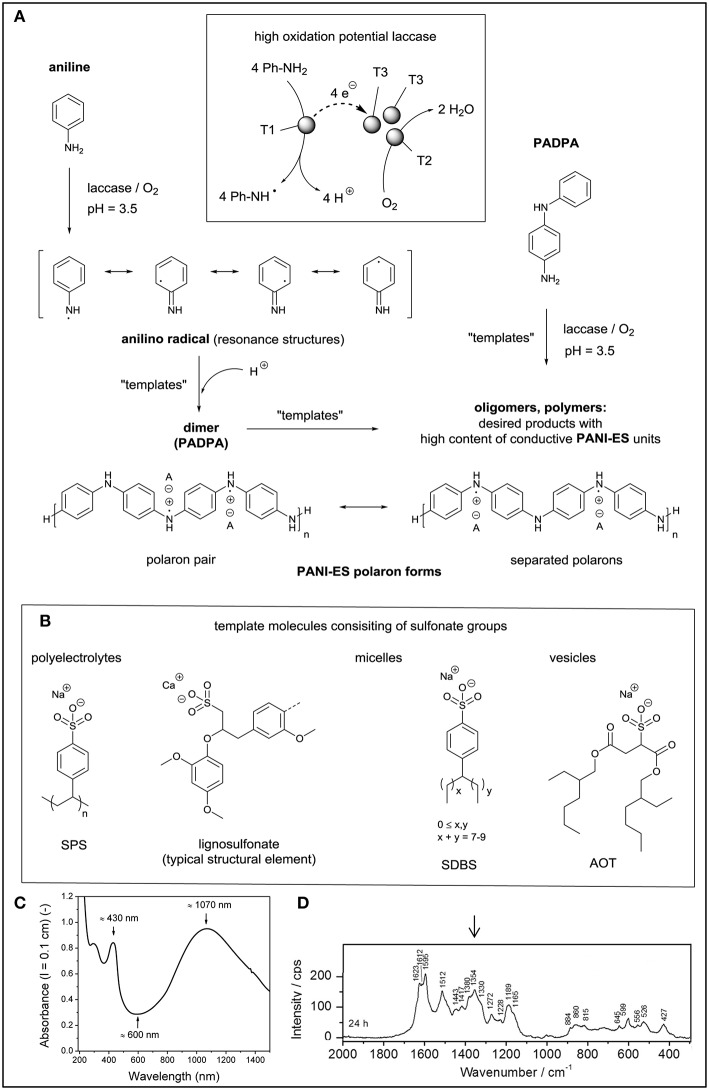
Template-assisted laccase/O_2_-catalyzed synthesis of oligomeric and polymeric products consisting of conductive PANI-ES units. **(A)** One-electron oxidation of aniline to the anilino radical, followed by the formation of the aniline dimer (PADPA) and polymeric products. Similarly, the use of PADPA as monomer can yield oligomeric PANI-ES-type products. **(B)** Some of the template molecules used, all containing sulfonate groups but differing in their state in aqueous solution: dynamic assemblies of polyelectrolytes, micelles, or vesicles. **(C)** Two examples of *in situ* recorded spectra of the obtained PANI-ES-type molecules formed with *Trametes versicolor* laccase/O_2_ from PADPA at pH = 3.5 in the presence of AOT vesicles as templates. Reproduced and modified from Kashima et al. (2019), with permission of the ACS. Further permissions related to **(B)** and **(C)** should be directed to the ACS.

If a high oxidation potential laccase is added to an aqueous solution containing aniline (Ph-NH_2_) as monomer in the absence of any mediator, the one-electron oxidation of the neutral form of aniline occurs in analogy to the oxidation of monolignols (see above) according to the following stoichiometric equation: 4 Ph-NH_2_ + O_2_ → 4 Ph-NH^•^ + 2 H_2_O (Figure 2A). This means that in each catalytic laccase cycle four aniline molecules are oxidized by the laccase at the expenses of one molecule O_2_, yielding four anilino radicals (Ph-NH^•^) and two water molecules as side products (Junker et al., 2014a). Ph-NH^•^ then undergoes follow-up reactions (radical-radical couplings and/or further oxidations). All follow-up reactions are probably no more under direct control by the enzyme, resulting in a mixture of products the composition of which being determined by the actual reaction conditions, i.e., the pH of the reaction mixture, the reaction temperature, the aniline concentration, the type and amount of laccase used, and whether “templates” are added or not. The term “template” stands here for any type of reaction additive which has a positive influence on the intended outcome of the reaction in terms of chemical structure of the reaction product(s) (Walde and Guo, 2011). This is somewhat related to the role “dirigent proteins” have in the biosynthesis of lignan and possibly also lignin (see above). For the enzymatic synthesis of electroconductive PANI, excellent templates are sulfonated polystyrene as sodium salt (SPS, Karamyshev et al., 2003), the calcium salt of ligninosulfonate (Zhang et al., 2016), micelles from sodium dodecylbenzenesulfonate (SDBS, Streltsov et al., 2009), or vesicles from sodium bis(2-ethylhexyl)sulfosuccinate (AOT, Junker et al., 2014a; Figure 2B). All these template molecules have sulfonate groups in their structures. In the presence of the templates and under optimal conditions (usually pH ≈ 3–4), the laccase/O_2_-catalyzed oxidation of aniline results in the formation of products which have the characteristic properties of the green conductive form of PANI, known as PANI-ES, the emeraldine salt form of PANI (Figure 2A). PANI-ES is the half oxidized, protonated form of linear PANI, with the aniline monomers coupled by N-C bonds in *para* position to the amino group. The repeating unit of perfect, defect-free PANI-ES is the half-oxidized, protonated linear tetraaniline shown in Figure 2A. Some of the characteristic properties of solutions or dispersions of PANI-ES in their conductive, polaron state are (Kashima et al., 2019): (i) high absorption intensity in the near infrared (NIR) region of the absorption spectrum, often with an absorption maximum at λ ≈ 800–1,100 nm, assigned to the π → polaron transition (do Nascimento and de Souza, 2015); (ii) an absorption band at λ ≈ 420 nm, assigned to the polaron → π^*^ transition (do Nascimento and de Souza, 2015); (iii) low absorption at λ ≈ 500–600 nm, indicative for the absence of extensive branching and phenazine unit formation (Liu et al., 1999b; Luginbühl et al., 2017); (iv) the presence of unpaired electrons, which results in an electron paramagnetic resonance (EPR) spectrum (Kulikov et al., 2002; Krinichnyi et al., 2006; Rakvin et al., 2014); and (v) characteristic Raman bands at ν ≈ 1,320–1,380 cm^−1^, originating from C–N^•+^ stretching vibrations of the polaronic form of PANI-ES (Ćirić-Marjanović et al., 2008a; Janoševic Ležaić et al., 2016).

The role of the templates during and at the end of the reaction has been investigated and discussed previously, mainly in reports of experiments using horseradish peroxidase isoenzyme C (HRPC) and added H_2_O_2_ as the oxidant (Liu et al., 1999a; Junker et al., 2012) instead of laccase/O_2_. The templates seem to direct the regioselectivity of the monomer coupling reaction due to a localization of the reaction in the vicinity of the templates, favoring *para*- over *ortho*-coupling of oxidized aniline. Furthermore, the templates act as dopants (counter ions) of the formed PANI (balancing the positive charge on the PANI polycation backbone), thereby stabilizing the PANI-ES structure which is essential for its property (electrical conductivity). This property is often the target of research on the laccase/O_2_- (or peroxidase/H_2_O_2_-) catalyzed polymerization of aniline, as environmentally friendly method, an alternative method to conventional chemical or electrochemical procedures (Stejskal et al., 2015). In the presence of good templates, the obtained PANI-ES products remain solubilized or dispersed in the aqueous reaction medium (no precipitation). Without template, but otherwise identical conditions, the formation of undesired, insoluble brown products is observed (Liu et al., 1999b; Guo et al., 2009).

It is very likely that in all studies that have been published on the laccase/O_2_-catalyzed oxidations of aniline in the presence of templates, *mixtures of different products* were obtained and not a single type of PANI-ES molecule with a fully defined chemical structure. This is due to the different follow-up reactions that may occur once anilino radicals (Ph-NH^•^) are produced by the laccase (see above). Unfortunately, due to the insolubility of at least some of the products obtained, a separation of all reaction products and an indisputable quantitative analysis of their chemical structures are impossible with current methods. Therefore, the overall product analysis mainly relies on *in situ* UV/vis/NIR, EPR, and cyclic voltammetry measurements and on a FTIR characterization of isolated product mixtures. The presence of the templates often assists in keeping the formed products dissolved or dispersed. This allows monitoring the formation of desired functional groups during polymerization and after reaching reaction equilibrium, for example by simple UV/vis/NIR measurements (Junker et al., 2014a). Interestingly, independent from the type of laccase and type of chosen template used, the UV/vis/NIR spectrum of the reaction mixture always appears to have an absorption maximum (λ_max_) between λ = 700 and 800 nm, and not at λ ≈ 800–1,100 nm, as expected for the π → polaron transition of PANI-ES (see above): λ_max_ ≈ 750 nm (with *Trametes hirsuta* laccase and SPS as template at pH = 3.5 and *T* = 20°C; Karamyshev et al., 2003); λ_max_ = 740–800 nm (with *Trametes hirsuta* laccase and SDBS micelles at pH = 3.8 and *T* = 20°C, Streltsov et al., 2008); λ_max_ ≈ 700 nm (with *Trametes hirsuta* laccase and poly(2-acrylamido-2-methyl-1-propanesulfonic acid) at pH = 3.5 and *T* = 20°C, Shumakovich et al., 2010); λ_max_ ≈ 750 nm (with *Trametes versicolor* laccase and κ-carrageenan at pH = 3.7 and room temperature, Leppänen et al., 2013); λ_max_ ≈ 750 nm (with Denlite®, a laccase preparation from *Aspergillus*, and SDBS micelles at pH = 4.5 and *T* = 10°C, Zhang et al., 2014); λ_max_ ≈ 750 nm (with Denlite® and lignosulfonate at pH = 3.5 and *T* = 5°C, Zhang et al., 2016); and λ_max_ ≈ 730 nm (with *Trametes versicolor* laccase and AOT vesicles at pH = 3.5 and T = 25 or 8°C, Junker et al., 2014a). These observed absorption maxima contrast with what has been observed for the HRPC/H_2_O_2_-catalyzed polymerization of aniline, even if the same template was used, e.g., AOT vesicles (λ_max_ ≈ 1,000 nm at pH = 4.3 and T ≈ 25°C, Junker et al., 2012; Pašti et al., 2017). One reason for this difference could be that the PANI-ES products which are obtained with laccase/O_2_ in the presence of a template are always in a “compact coil” conformation (MacDiarmid and Epstein, 1994, 1995; Yoo et al., 2007) with a shorter conjugation length and low delocalization of polarons, as compared to the PANI-ES products obtained in the presence of the same template with HRPC/H_2_O_2_; in the latter case, UV/vis/NIR spectra indicated an “extended coil” conformation and high delocalization of polarons (Xia et al., 1995). Alternatively, it may be that the PANI products obtained with laccase/O_2_ are overoxidized. This was the conclusion which was drawn in the case of PANI products obtained from aniline with *Trametes versicolor* laccase at pH = 3.5 in the presence of AOT vesicles as templates (Junker et al., 2014a).

One serious drawback of the template-assisted laccase/O_2_- or HRPC/H_2_O_2_-catalyzed polymerization of aniline is the relatively large amount of enzyme required for high aniline conversion. In the case of AOT vesicles as templates, for the oxidation of 1.0 g aniline, the estimated amount of pure *Trametes versicolor* laccase is also about 1.0 g (Junker et al., 2014a); in the case of HRPC, it is about 0.1 g enzyme (Junker et al., 2012). It is the formed polymeric product that inactivates the enzymes (Junker et al., 2012). From an economic point of view, such waste of enzyme is inacceptable, although the as obtained PANI-ES product may have excellent electrochemical properties, as shown for PANI-ES obtained with HRPC/H_2_O_2_ (Pašti et al., 2017). It is not clear at the moment how enzyme inactivation can be avoided or minimized for these reactions. Possible approaches toward an increase in operational enzyme stability could be to use mediator molecules for the reaction (Shumakovich et al., 2012a), to add polymers for stabilizing the enzyme (Junker et al., 2013), or to try to use immobilized enzymes (Vasil'eva et al., 2009).

## “Template” Effect on the Laccase/O_2_-catalyzed Oligomerization of PADPA

The aniline dimer PADPA (*p*-aminodiphenylamine, Figure 2A) is the first intermediate product which forms if two anilino radicals (or their protonated forms, i.e., aniline radical cations) react with each other in the desired way (head-to-tail coupling). PADPA must then undergo further reactions with aniline to finally yield linear PANI-ES. Due to this role as important intermediate product, PADPA has also been considered as monomer instead of aniline for the laccase/O_2_-catalyzed synthesis of PANI-ES. However, the reactivity of PADPA and the laccase/O_2_-catalyzed oxidation of PADPA differ considerably from those of aniline. First of all, PADPA is much easier to oxidize than aniline. The standard oxidation potential, ${\text{E}}_{\text{ox}}^{\circ }$ (PADPA) is about −0.4 to −0.5 V vs. NHE (Gospodinova and Terlemezyan, 1998), higher than in the case of aniline, ${\text{E}}_{\text{ox}}^{\circ }$ (aniline) = −1.0 V (Jonsson et al., 1994). Second, like in the case of the chemical or electrochemical oxidative polymerization of PADPA (Kitani et al., 1987; Geniès et al., 1989; Ćirić-Marjanović et al., 2008b), the majority of the products obtained from the laccase/O_2_-catalyzed oxidation of PADPA are *oligomers* and not true polymers (Shumakovich et al., 2011; Junker et al., 2014b; Luginbühl et al., 2016). This may have certain disadvantages in terms of applications, but it also has an analytical advantage. The obtained product mixture can be separated by high performance liquid chromatography (HPLC) with UV/vis diode array or mass spectrometry detection. This is at least the case for the products obtained from the *Trametes versicolor* laccase/O_2_-catalyzed oxidation of PADPA at pH = 3.5 in the presence (or absence) of AOT vesicles as templates (Junker et al., 2014b; Luginbühl et al., 2016; Kashima et al., 2018). Reaction product extraction into an organic solvent (*t*-butylmethylether) and analysis are possible for the specific reaction conditions used. They were optimized in terms of (i) high PADPA conversion, (ii) desired formation of PANI-ES-like products by using, (iii) low amounts of template, (iv) high colloidal stability, and (v) minimal amounts of enzyme. Before extracting the products into the organic solvent, they were deprotonated to make them soluble in the solvent. Afterwards, the products were reduced before applying on the HPLC column. After the entire product separation and identification of the molecules present, information on original protonation and oxidation states is lost. Only through the combination of the HPLC analysis—which was also carried out with partially deuterated PADPA monomers and ${\text{H}}_{2}^{18}$O—and complementary *in situ* UV/vis/NIR, EPR and Raman spectroscopy measurements of the entire reaction mixture recorded during the reaction and after reaching reaction equilibrium, clear conclusions about the effect of the AOT vesicle template on the outcome of the reaction could be drawn (Luginbühl et al., 2016; Kashima et al., 2018). Three essential findings are worth mentioning.

(i) The main product of the reaction in the presence of the vesicles is the *para*-N-C-coupled PADPA dimer in its half-oxidized, protonated state, i.e., the tetraaniline repeating unit of ideal PANI-ES (Figure 2A). This is the shortest possible PANI-ES type molecule and contributes most substantially among all formed products to the *in situ* recorded absorption spectrum of the reaction mixture, with λ_max_ ≈ 1,070 and ≈ 430 nm (Junker et al., 2014b; Kashima et al., 2018; Figure 2C), the *in situ* recorded EPR spectrum (Janoševic Ležaić et al., 2016; Kashima et al., 2018), and the *in situ* recorded Raman spectrum with characteristic band positions at ν ≈ 1,350–1,380 cm^−1^ (Janoševic Ležaić et al., 2016; Kashima et al., 2018; Figure 2D). Higher PANI-ES-type oligomers are also formed but to a much lesser extent.

(ii) If the reaction is run *without* template, many products contain an oxygen atom which originates from bulk water. The incorporation of the oxygen atom is most probably caused by hydrolysis of an intermediate diimine (the protonated form of *N*-phenyl-1,4-benzoquinonediimine). If the reaction is run in the presence of vesicles as templates, undesired products containing an oxygen atom are absent (Luginbühl et al., 2016).

(iii) Oligomers built from more than two PADPA molecules, some of them containing phenazine units, are formed in the presence as well as in the absence of vesicles, with the extent of phenazine formation being considerably higher without vesicles (Luginbühl et al., 2016).

In summary, the analysis of the *Trametes versicolor* laccase/O_2_-catalyzed oxidation of PADPA in aqueous solution at pH = 3.5 in the presence of anionic AOT vesicle templates has shown that the vesicles steer the reaction toward the formation of desired PANI-ES-like products. Certain undesired reaction pathways (hydrolysis or phenazine formation) are prevented or largely avoided. Although the detailed molecular picture is still fragmentary and current mechanistic hypotheses still need to be proven or disproven, it is clear that the reproducible preparation of PANI-ES-like oligomers, which have the characteristic spectroscopic features of chemically synthesized PANI-ES, is possible under environmentally friendly conditions with laccase/O_2_ as efficient catalyst, PADPA as monomer, and an appropriate template. Apart from AOT vesicles, other templates with sulfonate groups can be used as well, SPS polyelectrolyte, SDBS micelles or SDBS/decanoic acid (1:1) vesicles, see Kashima et al. (2019). For each template, optimal reaction conditions have to be elaborated. Furthermore, the template type has an influence on the property of the obtained mixture of products (Kashima et al., 2019).

## Conclusions

Although there is no doubt that high oxidation potential laccases can catalyze the oxidation of aniline or its dimer PADPA in aqueous media in the presence of dissolved O_2_, the outcome of the reaction depends on many factors. The presence of an anionic template is essential for obtaining the emeraldine salt form of oligo- or polymeric aniline products. This template effect is related to the role “dirigent proteins” have in the biosynthesis of lignin. Since the polymeric products obtained from aniline are difficult to isolate and analyze individually—often despite their promising electrochemical properties—the oligomeric products obtained from PADPA allow gaining insight into the effect the templates exert on the reaction. The templates act as soft, dispersed interface-rich additives (Serrano-Luginbühl et al., 2018) that guide the reactions toward desired products. Despite uncertainties in the composition and chemical structure of the reaction products obtained, the best choice of monomer, laccase type, and template may depend on the actual product application in mind. Nevertheless, the challenge remains in improving the knowledge about the guiding effect of the template—also for other related reactions (Junker et al., 2015)—so that the reaction conditions can be tuned in a controlled way for achieving products with desired properties in high yield under as environmentally friendly conditions as possible.

## Author Contributions

PW outlined the article. All authors contributed to the writing.

### Conflict of Interest Statement

The authors declare that the research was conducted in the absence of any commercial or financial relationships that could be construed as a potential conflict of interest.
